# Why Victory in the War on Cancer Remains Elusive: Biomedical Hypotheses and Mathematical Models

**DOI:** 10.3390/cancers3010340

**Published:** 2011-01-17

**Authors:** Leonid Hanin

**Affiliations:** Department of Mathematics, Idaho State University, 921 S. 8^th^ Avenue, Stop 8085, Pocatello, ID 83209-8085, USA; E-Mail: hanin@isu.edu

**Keywords:** angiogenesis, breast cancer, cancer dormancy, mathematical model, metastasis, model identifiability, poisson process

## Abstract

We discuss philosophical, methodological, and biomedical grounds for the traditional paradigm of cancer and some of its critical flaws. We also review some potentially fruitful approaches to understanding cancer and its treatment. This includes the new paradigm of cancer that was developed over the last 15 years by Michael Retsky, Michael Baum, Romano Demicheli, Isaac Gukas, William Hrushesky and their colleagues on the basis of earlier pioneering work of Bernard Fisher and Judah Folkman. Next, we highlight the unique and pivotal role of mathematical modeling in testing biomedical hypotheses about the natural history of cancer and the effects of its treatment, elaborate on model selection criteria, and mention some methodological pitfalls. Finally, we describe a specific mathematical model of cancer progression that supports all the main postulates of the new paradigm of cancer when applied to the natural history of a particular breast cancer patient and fit to the observables.

## 1. Introduction

In 1971, President Richard M. Nixon declared the “War on Cancer.” Since then, cancer research and treatment are typically described in combative terms: scientists are depicted as “cancer fighters,” patients “battle with cancer,” and those who succumb to the disease are likened to fallen soldiers. This reflects, perhaps subliminally rather than consciously, the traditional view of cancer as an adversarial entity, akin to pathogenic bacteria or viruses, seeking to destroy the human organism. This image of cancer has also informed therapeutic strategies: if cancer is an external foe, it should be killed through aggressive treatment. The question, then, becomes: are we approaching victory in the war on cancer, and if not, then why?

Recent decades have witnessed an unabated increase in the incidence of several major types of cancer and, most alarmingly, an upward trend, relative stabilization or only moderate decline in cancer-specific mortality (with some exceptions, most notably, breast cancer). In spite of dramatic progress in the treatment of several cancers—such as thyroid cancer, Hodgkin lymphoma, and acute lymphocytic leukemia in children—there has been only limited progress in treating those types of cancer that claim by far the largest toll of human life, including cancer of the breast, prostate, lung, liver, kidney, and colorectal cancers. Incidentally, these solid cancers are not lethal *per se*, unless the primary tumor reaches a size so large as to threaten the functioning of the affected organ or other vital organs; rather, they mostly kill through metastases that are responsible for about 85–90% of all cancer-related deaths.

Advancement of biomedical and physical sciences over the last 120 years has dramatically changed the landscape of clinical oncology. Due to the progress in detection and imaging technology and discovery of various histological, biochemical, and genomic markers, cancer can be detected at much earlier subclinical stages. Rapid progress in radiation biology and associated medical physics led to new modes of cancer radiotherapy, including brachytherapy, radioimmunotherapy, and conformal stereotactic radiosurgery. Also, novel technologies of external beam radiation allow oncologists to deliver safely considerably larger doses—currently up to 10 Gy—to a specific site of interest, while largely sparing healthy tissue. The advent of the era of molecular biology has brought about new chemotherapeutic agents that have cytotoxic, anti-angiogenic, and hormone-blocking activity. Finally, modern standards of cancer patient care are incomparably higher than those in the recent past. Yet, in the grand scheme of things, the progress has been largely incremental and, most tellingly, the rates of tumor recurrence and metastatic relapse have essentially saturated at levels that give no grounds for optimism.

One would think that this should have triggered a major concern in the biomedical community, and a call to action. Privately, many have expressed such concerns, which have occasionally spilled over into the mass media [1]. By and large, however, inertia of thought, science politics, and vested interests have prevented a concerted, critical examination of the reasons why great advances in modern biology and exorbitant investments in the public health complex over the last 40 years have not yielded revolutionary breakthroughs in the understanding and treatment of cancer. For the same reasons, many groundbreaking ideas advanced over recent decades by a number of scientists and medical doctors have not so far enjoyed broad recognition and adequate testing. In particular, this applies to a new paradigm of cancer progression and treatment that was developed since the mid 1990s by Michael Retsky, Michael Baum, Romano Demicheli, Isaac Gukas, William Hrushesky and their co-workers based on the earlier pioneering work of Bernard Fisher and Judah Folkman.

In this article, we discuss philosophical, methodological, and biomedical grounds for the traditional paradigm of cancer and some of its critical flaws. We touch upon various reasons for the persistence of the traditional paradigm of cancer, in spite of profuse epidemiological, clinical, and experimental evidence pointing to its inadequacy. We also review and systematize some potentially fruitful alternative approaches to understanding cancer and its treatment, including the aforementioned new paradigm of cancer. Next, we highlight the unique and pivotal role that mathematical modeling can play in testing biomedical hypotheses about the natural history of cancer and the effects of its treatment, discuss relevant methodology, elaborate on model selection criteria, and mention some methodological pitfalls. As an example, we describe a specific mathematical model of cancer progression that supports all the main postulates of the new paradigm of cancer when applied to the natural history of a particular breast cancer patient and fit to the observed volumes of bone metastases. Finally, we discuss how the model can be refined and expanded to be applicable to other patients, treatment regimens, and cancer types and to uncover quantitative aspects of the mechanisms postulated by the new paradigm of cancer.

## 2. The Grand Strategy of the War on Cancer: What is Wrong?

If a war effort fails to bring victory, the first place to look for the roots of failure is the military doctrine. Accordingly, we start with examination of the dominant paradigm of cancer.

### 2.1. Genetic Determinism vs. the Role of Microenvironment

Most theories of carcinogenesis and cancer progression center on genetic determinism. It posits that

1. cancer is brought about by a sequence of mutations; some mutations may be hereditary, while others can occur spontaneously, or be induced by mutagenic chemical carcinogens or radiation; and
2. the natural history of cancer is essentially genetically driven.

What this theory of cancer tends to neglect is a large body of biological and clinical evidence, both old and modern, suggesting that cancer exists in a state of dynamic homeostasis resulting from close interaction with its microenvironment, cells and proteins of the immune system, and hormones. The interaction of a tumor with its environment involves recruitment of cells from the surrounding stroma, exchange of signals, as well as competition for nutrients, growth factors, and access to the capillary network. Within this framework, cancer can be thought of as a tissue or organ-like entity that has switched to an alternative functional pathway (newly acquired or pre-existing in a latent form) that causes cancer cells to change their proliferative behavior and to gain the ability to block or evade apoptotic signals, inhibit ligands of death receptors, migrate, degrade extracellular matrix, invade surrounding tissues, and metastasize.

Evidence for the fundamental role of microenvironment in tumor dynamics comes from many directions:

- In the classic experiments conducted by Beatrice Mintz and Karl Illmensee in 1975, teratocarcinoma cells were implanted into murine embryos at a very early stage of embryonic development [2]. In spite of this, the embryos gave rise to perfectly normal mice. In another set of experiments by David Dolberg and Mina Bissell [3], the Rous sarcoma virus caused a tumor when injected into the wings of chickens, but it failed to do so when injected into the embryos. These experiments should prompt us to supplement the view of cancer as a genetically driven progressing pathology with the notion that cancer might be a reversible phenotype. Uncovering the conditions of tumor microenvironment under which a particular type of cancer can be reversed might provide a window of opportunity for radical cure.
- An even more heterodox thesis—that cancer is a disorder of cellular organization—was argued by Sir David Smithers [4]. In his own words, “cancer is no more a disease of cells than a traffic jam is a disease of cars.”
- It has been observed since time immemorial that metastases shed by certain types of tumors only surface in specific secondary sites (such as liver, bones, lungs, brain, soft tissue, etc.) and are very rarely found in other organs or tissues. In 1889, Stephen Paget explained this selective affinity of metastases to certain sites by their sensitivity and adaptation to the conditions of host microenvironment (“seed and soil hypothesis”) [5].
- The role of tumor microenvironment in carcinogenesis sheds some light on the profuse evidence, albeit mostly anecdotal, that primary cancer may be triggered by a trauma, bacterial infection, or a virus not directly related to the cancer in question.
- Spontaneous regression of various types of cancer, most notably, adenocarcinoma of the kidney, neuroblastoma, melanoma, choriocarcinoma, carcinoma of the bladder, and sarcomas of bones and soft tissue, is a well-documented phenomenon, see e.g. early monographs [6,7]. More recently, a 22% rate of spontaneous breast cancer cure was inferred from a large-scale study involving more than 229,000 women followed from 1992–2001 in Norway [8]. Clearly, much greater is the wealth of cases where cancer regression goes undocumented. It can be hypothesized that tumor microenvironment—along with the immune response—plays a major role in bringing about spontaneous cure.
- While opinions on this subject vary, breast cancer progression may be modulated by menstrual, seasonal, and circadian cycles that can have significant effects on tumor microenvironment [9-11]; see also review [12] and references therein.

### 2.2. Linear Progression of Cancer

The idea that temporal progression of cancer is inextricably linked to spatial, or anatomic, spread of the disease has become a central dogma of oncology. Reinforced by the classification of cancer stages as localized, regional, and distant (with a number of intermediate stages) it has shaped, and continues to shape, the clinical thinking of several generations of medical doctors. This is one of the reasons why early seminal work pointing to the folly of the linear progression theory [13], as well as more recent ample evidence against this theory, has not led so far to a revision of this cornerstone of oncology.

The theory of linear cancer progression has two important implications for cancer treatment: (1) cancer should be diagnosed and treated as early as possible; and (2) the later the cancer stage at primary diagnosis the greater the extent of treatment required. In the case of breast cancer, this approach has produced two noteworthy upshots: first, a widespread and costly mammographic screening effort; second, a scale of increasingly aggressive surgery options commensurate with the stage at primary diagnosis (lumpectomy, mastectomy, and mastectomy combined with excision of breast wall muscles and proximal lymph nodes) and augmented, when necessary, with adjuvant radio-, chemo-, and/or hormonal therapy. On the prostate cancer front, this approach has been paralleled by aggressive advocacy for prostate-specific antigen (PSA) screening, prostatectomy with a number of surgical extensions, and an assortment of adjuvant treatments including external beam radiation or brachytherapy.

The reality of cancer treatment outcomes, however, diverged from the expectations in many different ways:

- On the grounds of linear progression theory it was believed that very early extirpation of the primary tumor would result in a permanent cure. In reality, a significant fraction of patients diagnosed and treated at the earliest stages of cancer later relapsed with distant metastatic disease.
- The pioneering research of Bernard Fisher conducted in the 1970s and 1980s showed that, in terms of overall survival, mastectomy is as effective as more extensive surgery involving chest wall and axillary lymph nodes, while lumpectomy followed by radiation does not worsen the overall probability of survival compared to mastectomy alone.
- The linear theory of cancer progression made early cancer detection all the rage. Starting in the 1970s, mammographic screening for breast cancer (sometimes combined with a physical exam) was advocated vigorously and became widely used. To ascertain the efficacy of mammography, large scale clinical trials were conducted in the U.S., Canada, and a number of European countries in the 1970s-1990s. After the dust of the battle over interpretation of the results of these trials had settled, the picture that emerged showed that the overall utility of mammography is uncertain [14]. For every life saved due to early detection of lethal breast cancer, 10 women are treated unnecessarily for cancer that is not life threatening or may go away on its own. Most surprisingly, however, for a particular category of women (specifically, node-positive pre-menopausal women aged 40–49) mortality rates for 6–8 years post-mammography actually increased. This finding was one of the most striking clues that something is amiss in the linear progression theory. A very recent study of breast cancer mortality trends in Europe revealed that the decline in mortality was by far the largest among women younger than 50 years old, an age group that is very rarely screened in Europe [15]. This proves beyond reasonable doubt that mammography has only moderate effect, if any, on breast cancer mortality. The mammography conundrum had a profound impact on the medical world and society at large. As a result of it, mammography screening was considerably scaled down in 2009 by the U.S. Preventive Services Task Force (the starting age was increased from 40 to 50 years, annual exams were replaced with biannual ones, and overall mammography recommendation became less forceful, except for women with familial history of breast cancer or those carrying certain genes).
- The story of PSA screening is no prettier. After the discovery of PSA and development of the relevant blood test (typically combined with digital rectal exam of the prostate) PSA became a gold standard for tumor markers [16]. But very much like mammography, PSA screening led to massive overdiagnosis, combined with failure to timely detect deadly tumors, decreased survival in a subset of cases, and reduced quality of life for those unduly treated. In fact, one death of prostate cancer prevented by early diagnosis through screening comes at a cost of about 50 men needlessly treated for relatively innocuous tumors. Although American men older than 50 years of age have a 16.7% probability of developing prostate cancer, the probability to die from it is only 3.6% [17]. Moreover, autopsy studies show that 30–40% of men will develop cancerous foci in the prostate which, in most cases, will never reach the clinical stage during their lifetimes [18]. In the words of no less than Richard Ablin, who discovered PSA in 1970: “I never dreamed that my discovery four decades ago would lead to such a profit-driven public health disaster. The medical community must confront reality and stop the inappropriate use of P.S.A. screening. Doing so would save billions of dollars and rescue millions of men from unnecessary, debilitating treatments” [19].
- In the times of antiquity and the Middle Ages many prominent physicians were of the opinion that surgery on breast cancer precipitates death of patients unless given very early [20]. One can argue, of course, that they did not know what we know now. History shows, however, that in great individuals, a lack of knowledge can be offset by sharp intuition based on extensive experience and a well-honed capacity for careful observation.

### 2.3. Interaction between Primary and Secondary Tumors

The paradigm of a tumor as an autonomously growing entity whose natural history is determined by its evolving genotype and constrained by the availability of nutrients, oxygen, and growth factors has had another important consequence: consistent neglect of the interaction between primary and secondary tumors. On this front, more than a century of experiments on animals and clinical observations on humans, reviewed at length in [12,20], has brought forth a wealth of striking findings. Here is a brief summary:

- All tumors, both primary and secondary, inhibit each others' growth. The degree of the inhibiting effect that tumor A has on tumor B increases with the size of tumor A, so that the largest tumor exerts the greatest inhibiting effect on other tumors. In particular, the growth of metastases in the presence of primary tumor saturates at lower levels than in its absence. The first discovery of this kind was made by the great Paul Ehrlich and his assistant Hugo Apolant whose extensive tumor graft animal experiments conducted in the early 1900s at the Ehrlich's Serum Institute in Frankfurt am Main showed that earlier tumors suppressed the growth of tumors injected later [21].
- In animal experiments, tumor bearing subjects were found to be refractory to inoculation with a dose of tumor cells several orders of magnitude higher than the dose required to produce a primary tumor. Qualitatively similar effect was observed in humans (in studies involving incurable patients).
- In some experiments, average life expectancy of mice with two tumors was higher than with one.
- An important role in limiting the growth of secondary tumors in the presence of the primary is played by angiogenesis inhibitors, such as angiostatin and endostatin [22].
- The first explanation of these phenomena was suggested by Richmond Prehn [23] in 1993. Later it was elaborated and expanded in a series of works by Judah Folkman, Michael Retsky, Michael Baum, Romano Demicheli, Isaac Gukas, William Hrushesky and their colleagues [12,20,24], and became a compelling working hypothesis that explains a broad range of clinical findings. According to this hypothesis, tumor dynamics is determined by the interplay between production and disintegration of the factors that stimulate or inhibit growth and angiogenesis. All of them are produced by tumors and their microenvironment. However, while growth and angiogenesis factors have relatively short lifetimes and spread mostly locally by diffusion, the inhibitors are more stable, and, when released into the bloodstream, may reach other primary or secondary tumors and impede their growth.

## 3. Cancer Diagnosis and Prognosis: Fundamental Problems Ignored

The rapid advancement of molecular biology, biochemistry, genomics, and bioinformatics in recent decades has led to a dramatic redistribution of intellectual and financial resources in favor of these flourishing fields of knowledge. They satisfy intellectual curiosity, impel careers, and promise lavish funding. Yet many exciting developments in modern biology have not translated into a revolution in cancer treatment. Undoubtedly, developing new drugs and therapies, testing them, and obtaining approval take time. However, progress in another vital area—cancer diagnosis and prognosis—that one can expect to be an immediate beneficiary of advances in basic biology, has also been far from revolutionary. In fact, a number of questions forming the very core of clinical oncology have been largely ignored.

### 3.1. Uncertainty in Pathology Readings

Any suspicious finding that results from cancer screening leads to a more extensive study, most likely a biopsy. In the case of breast cancer, this produced an increased caseload of pathology studies aimed at differentiation between invasive breast cancer, ductal carcinoma *in situ* (DCIS), and a number of benign conditions, such as fibroid tumor, atypical ductal hyperplasia or a cyst. It became apparent that histology and pathology are not advanced enough to establish a reliable diagnosis at such an early stage of the disease. In a large fraction of cases pathologists disagree on the same slide of breast tissue. Moreover, there are even different schools of thought as to what constitutes DCIS. As a result, a significant number of cases are either over- or underdiagnosed. This has triggered various corrective measures by the College of American Pathologists, and an outcry in the media [25] documenting several cases where women underwent disfiguring surgery, radiation, and chemotherapy, only to learn later that they did not have breast cancer. Finally, in the case of DCIS or invasive breast cancer, a critical question as far as treatment options are concerned is whether cancer will surface and become life threatening during the patient's lifetime. The answer is not even in the offing.

### 3.2. The Clonogenicity Problem

Along with actively proliferating cells, termed *clonogenic*, a tumor contains slowly proliferating and quiescent cells that are essentially clonogenically dead. Furthermore, larger tumors contain a hypoxic core consisting of quiescent cells and proteins from cells that underwent lysis, while clonogenic cells are predominantly concentrated at the tumor periphery, closer to the capillary network.

Clonogenic capacity of a cell can be quantitatively assessed in an explantation assay by measuring the size of the clone produced by a progenitor cell in a growth stimulating medium. Of course, such clonogenic capacity is hardly informative of the clonogenicity of the cell *in vivo* due to various effects of intratumor interactions and the influence of tumor micro- and macroenvironment. Therefore, clonogenicity of a tumor cell remains largely unobservable. Accordingly, estimates of the fraction of clonogenic cells in a solid tumor reported in the literature vary from a tiny fraction of 1% to numbers close to 100% and thus remain largely speculative. Primary tumor volume at diagnosis, then, is a weak, perhaps even misleading, prognostic clinical variable. It is perplexing that no *in vitro* clonogenic capacity tests—histological, biochemical, or genomic—have been developed so far. A lack of such tests is a major impediment to progress in cancer treatment, for it is the subpopulation of clonogenic cells that is the real target of any cancer therapy, and most likely, it is also the main source of metastases.

Understanding and measuring clonogenicity also seems to be a key to rationalizing the somewhat elusive concept of “cancer stem cell”. The latter may well be merely a label for cells with very high clonogenic capacity. The fact that some cancers appear to have “cancer stem cells” while others do not can be explained by the presence or absence of a small fraction of extremely clonogenic cells within the bulk of a tumor.

### 3.3. Tumor Heterogeneity

Varying clonogenicity of tumor cells is only one of many facets of tumor heterogeneity. Another important aspect of variability of cancer cells is the presence of fast and slowly growing subpopulations of clonogenic cells. Ideally, the fastest growing cells should have the highest priority for elimination. An additional dimension of tumor heterogeneity that is directly related to the outcome of treatment is the variation of sensitivity of tumor cells to a particular therapy. Essentially, it is the size and proliferation rate of the most resistant subpopulation that determines the outcome of any protracted therapy. We note finally that sensitivity to treatment is not directly related to the rate of proliferation and clonogenicity. For example, hypoxic cells typically have low clonogenicity and are slowly proliferating or quiescent, yet they are the most radioresistant.

### 3.4. Selection Effects

Another poorly understood factor that exerts a major influence on treatment outcomes is various selection effects induced by treatment. As one example, imagine that before treatment, a tumor consists of two subpopulations of equal size, one of them growing at a rate twice as fast as the other subpopulation. It is natural to assume that none of the two subpopulations has survival advantage, so that at the end of treatment there remains, say, only 1% of cells in each subpopulation. Suppose that during the post-treatment regrowth phase, both subpopulations grow exponentially with the pre-treatment growth rates. Then, an easy computation would show that at the time the tumor reaches its pre-treatment size, faster growing cells will constitute more than 90% of the tumor! This explains why each next round of treatment has less favorable chance of success and leads to faster relapse, unless the tumor is completely eradicated.

Similar selection effects are applicable to other characteristics of malignant cells that impact tumor dynamics through rates of proliferation, spontaneous or treatment-related death, or cell cycle duration. For example, if cell sensitivity to treatment is unrelated to the rate of cell proliferation, then in the course of treatment the tumor population becomes increasingly dominated by resistant cells.

### 3.5. Metastatic Potential of a Tumor: The Elephant in the Room

When a patient is diagnosed with invasive cancer, the question of ultimate importance on which selection of treatment hinges is this: What are the probabilities that metastases will surface during the patient's lifetime under a given treatment plan and without treatment? Any attempt to answer this question requires an assessment of a tumor's metastatic potential.

To form a detectable metastasis in a secondary site, a malignant cell has to separate itself from the primary tumor, migrate toward a blood or lymph vessel, overcome extracellular matrix barriers, intravasate, traverse the circulatory network, evade attacks of the immune system, extravasate, invade a host site, survive through the dormancy period, start to proliferate, and induce angiogenesis. As a result of this multi-stage selection process, only a tiny fraction of cells seeded by the primary tumor gives rise to actively growing metastases [26]. How is metastatic potential of a malignant cell related to its clonogenicity? What are the genotypes of tumor cells with high metastatic potential? How large is the fraction of such cells in the tumor? Do these cells preexist in the tumor or emerge as a result of mutation, selection, adaptation, and interaction with the environment? Answers to these questions are the primary determinants of the decision on whether a particular tumor should be treated (and by which means) or watchfully observed. Resolving these questions constitutes the Holy Grail of oncology; yet the biomedical knighthood has not shown much zeal in pursuing it.

## 4. The Emerging New Paradigm of Cancer and Its Treatment

The inadequacy of the traditional paradigm of cancer had become clear long before enough scientific evidence against it was collected. In fact, even a predominantly philosophical analysis of some of its foundations generates enough skepticism with regard to their validity, see Section 2. Building a new paradigm of cancer, however, was a long and gradual process. The main idea is that cancer is a systemic disease starting with its very early subclinical stages. This includes early dissemination of metastases; their dormancy as single cells; dormancy or slow, saturated, avascular growth of small clumps inhibited by the primary tumor; and abrupt acceleration of metastatic growth and angiogenesis that brings about a sudden and unbridled exacerbation of the disease due to resection of the primary tumor or any other disruption of the balance between growth stimulating and inhibiting agents as well as pro- and anti-angiogenic factors and signals.

Many scientists and physicians highlighted various aspects of this paradigm: Bernard Fisher (systemic nature of cancer and its early dissemination); Richmond Prehn (interaction between primary and secondary tumors); Judah Folkman (the role of angiogenesis and mechanisms of angiogenic switch), and many others. However, the new conceptual scheme, mostly focused on breast cancer, started to crystallize only in the works of Michael Retsky, Michael Baum, Romano Demicheli, Isaac Gukas, William Hrushesky and their colleagues published since the mid 1990s and reviewed at length in [12,20]. This created an alternative paradigm of cancer that successfully explains a large body of epidemiological data, clinical observations, and experimental findings many of which have heretofore eluded alternative explanations.

The initial impetus to the new paradigm of cancer came from the analysis of epidemiological data on breast cancer cure, relapse, and mortality, see [20,24] and references therein. The new paradigm of breast cancer predicts and explains a variety of seemingly unrelated biomedical findings, such as (1) increased mortality of pre-menopausal node-positive women mammographically diagnosed with breast cancer at age 40–49 in a number of large-scale clinical trials, combined with increased survival for other categories of women (the “*mammography paradox*”) [20,27,28]; (2) the fact that adjuvant chemotherapy is more beneficial to pre-menopausal node-positive women afflicted with breast cancer than to other categories of breast cancer patients [29]; (3) similarity in temporal patterns of local and distant breast cancer recurrence irrespective of the recurrence site [30]; and (4) very low incidence of breast cancer in women with Down syndrome [31].

Paradoxically, the new paradigm of cancer emerged not from the latest advances of molecular biology and genetics but rather from careful analysis of epidemiological data, a relatively small number of decisive animal experiments, and through thoughtful reading of a large body of clinical and experimental works published over the last 140 years, with many clinical observations tracing back to Middle Ages and antiquity [12,20]. Below we review very briefly the content and empirical basis of the hypotheses that constitute the new paradigm of cancer, emphasizing their relevance to many types of cancer.

### 4.1. Early Metastatic Dissemination

The hypothesis that metastatic dissemination of invasive cancer may start long before primary tumor becomes clinically detectable has been entertained for decades [13,26], see also reviews [12,20] and references therein. For example, it was estimated in [26] that more than 70% of cancer patients have occult metastases at presentation. This suggests that metastatic dissemination and invasion are very early events in the natural history of cancer. In fact, metastases can spread to local lymph nodes as soon as primary tumor cells gain competence in migration and penetration of lymph channels. Similarly, bloodborn metastases may take root in distant organs and tissues as soon as primary tumor develops a capillary network.

The early metastasis hypothesis gained a substantial clinical grounding in the path breaking works of Bernard Fisher [32]. He predicted that the extent of local treatment would have only limited effect on long-term survival and cure, which was confirmed later by numerous clinical trials. He also proposed that systemic treatment for breast cancer should be started even in the absence of nodal involvement and clinically manifest distant metastases.

An unequivocal confirmation of the hypothesis of early metastatic spread of cancer would indeed suggest that certain categories of cancer patients should be treated for metastases concurrently with, or shortly after, the treatment of the primary tumor. The emerging new methods of metastases ablation such as conformal stereotactic radiosurgery (designed for treatment of isolated clinically manifest metastases), radioimmunotherapy and treatment with oncolytic viruses (suitable for inactivation of both detectable and occult metastases of any size and localization), and other modes of targeted therapy make such an approach to treatment potentially feasible.

### 4.2. Circulating Tumor Cells

The first report of circulating tumor cells (CTCs) dates back to as early as 1869 [33]. The “seed and soil” hypothesis proposed by Stephen Paget in 1889 [5] was a response to this and other similar findings, and represented an attempt to explain why many cancer cells keep circulating in the blood rather than spawning metastases and why metastases shed by a primary tumor only surface at some specific secondary sites while other sites remain metastasis-free. The CTC phenomenon was later confirmed by numerous modern studies. It was shown in the cases of breast cancer [34], lung cancer [35,36], melanoma [37], and esophageal cancer [35] that large quantities of CTCs can be present without overt metastases. It was found that about one-third of breast cancer patients 7–22 years after mastectomy and without any evidence of the disease had CTCs [38]. The presence of CTCs in peripheral blood was also confirmed in 24% of prostate cancer patients before prostatectomy [39] as well as in patients with undetectable PSA levels more than five years post-prostatectomy [39,40].

The prevalence of CTCs calls for a detailed study of this phenomenon. One of the sources of CTCs is undoubtedly undetected primary and occult secondary tumors that continuously shed their cells into the circulatory system. Another possibility is that a fraction of cancer cells circulating in blood and lymph vessels could remain dormant during a protracted period of time.

### 4.3. Cancer Dormancy

The concept of cancer dormancy was first introduced in 1954 [41]. Since then it has changed its status from an exotic hypothesis to one of the central themes in cancer research, as evidenced by a recent NCI workshop dedicated entirely to this subject [42]. Dormant cancer may exist in several forms: dormant malignant cells at the site of the treated primary tumor; quiescent solitary metastatic cells; and dormant or slowly growing secondary tumors. In the latter case, dormancy is maintained through a dynamic balance between cell proliferation and death due to apoptosis, influence of the microenvironment, and the inhibiting effect of the primary tumor. The evidence for cancer dormancy is manifold:

- Reports on breast cancer recurrence 20–25 years after treatment of the primary tumor [43].
- Autopsy studies revealing dormant malignant cells and microscopic cancerous foci in many organs and tissues.
- Direct observations of dormant cancer cells at various sites through *in vivo* video microscopy.
- Systematic study of the conditions under which dormancy of hepatic metastases can be maintained or interrupted, which results in their active proliferation, in animal models [44].
- Discovery of dormant prostate cancer cells in the bone marrow of 44% patients in a group of patients who underwent prostatectomy and had undetectable PSA levels more than five years post-treatment [40,42].
- An indirect confirmation of dormancy in breast cancer coming from the observed bi-modality of the hazard rate of time to distant recurrence [45], which is incompatible with the hypothesis of continuous growth of secondary tumors.
- The fact that the preponderance of CTCs in disease-free patients over long periods of time cannot be explained without assuming occult dormant tumors that continuously shed cancer cells into the bloodstream.
- Reports of the transmission of dormant metastases to the recipients of organ transplants. In one case, metastatic prostate cancer cells were transmitted through a heart transplant from a donor, whose autopsy confirmed prostate cancer, to a recipient, whose prostate was free of the disease, but who developed a prostate metastasis in a rib [46]. This case suggests that dormant cancer cells can exist in organs (such as heart) that do not normally support their growth. In another case, a glioblastoma multiforme metastasis developed in a patient's liver transplanted from a donor who died of this same type of malignancy [47].

The clinical significance of metastasis dormancy cannot be overstated. It explains the failure of local treatment in a large fraction of cancer patients regardless of the stage of the disease, and necessitates an overhaul of cancer treatment strategies. On the positive side, it suggests a less radical but perhaps more realistic and practical approach to cancer treatment—in place of fighting cancer just to live with it by maintaining the state of dormancy. To implement this approach, one has to learn how to target dormant, rather than fast proliferating, cancer cells.

### 4.4. Angiogenesis and Cancer Progression

It was known for centuries that, like any growing or regenerating organ, primary or secondary tumor develops a network of blood vessels that grow out of the existing vasculature and supply the tumor with nutrients and oxygen. But it took the genius of Judah Folkman to uncover the discontinuous nature of this process and to discern here an opportunity for novel cancer treatment. He showed that, in the absence of capillary network, a tumor, in spite of its high proliferative capacity, can grow only up to a certain microscopic size, and remain in this state for a protracted period of time. A key condition for maintaining this state of equilibrium is the balance of angiogenesis-promoting and inhibiting factors and signals. When the balance is disrupted due to trauma, wounding, infection, irradiation, excision of the primary tumor or any other surgery, angiogenesis is activated, which causes irreversible, aggressive tumor growth.

Folkman developed the blueprint of this revolutionary approach in the early 1970s. His idea had little to do with genes and molecular biology and, consequently, was out of sync with the academic fashions of the day. Rather, angiogenesis and “angiogenic switch” depend on much simpler and perhaps more fundamental phenomena: chemical gradients, biochemical kinetics, cell motility, chemotaxis, and fractal geometry of capillary networks. As a result, Folkman's ideas were kept out of the mainstream, and even ridiculed, for almost a quarter century. This notwithstanding, he doggedly pursued his vision and brought it to fruition. In particular, his insights led to the development of effective anti-angiogenic drugs like Avastin that gave reprieve to millions of cancer patients.

### 4.5. Surgery Induced Accelerated Growth of Metastases

The uncovered interaction between primary and secondary tumors (see Section 2.3) has far-reaching implications for cancer control and treatment. The most prominent among them is the effects of surgical removal of the primary tumor on metastases. Resection of the primary tumor is followed by an abrupt drop in the rate of production of inhibitors of growth and angiogenesis. Concurrently, local and systemic wound healing processes produce a surge in the concentration of growth and angiogenesis factors. The combination of these processes breaks a delicate balance between stimulation and inhibition of growth and angiogenesis. This interrupts the state of dormancy of metastases or their slow avascular growth, and results in aggressive proliferation and vascularization of secondary tumors. Because radiation brings about qualitatively similar dynamics of growth and angiogenesis factors and their inhibitors, it can be hypothesized that it has a similar effect on metastatic progression.

It is well-known that most cancer patients, even those with large primary tumors, do not have clinically manifest distant metastases at presentation; yet in a large fraction of those patients, distant metastases emerge within months or several years after surgery. How could this elementary observation not have triggered a careful look at the effects of surgery on metastatic progression of cancer many decades ago? This is even more inexplicable given that experimental evidence that primary tumor resection can accelerate growth of metastases in animal models has first emerged as early as in 1910s, and kept on accumulating, and being confirmed through clinical observations and epidemiological studies, ever since, see [12,20,24] for an extensive review. Furthermore, it became evident that wounding unrelated to the primary tumor excision [48], and even biopsy [49], produce similar effects. The boosting impact of primary (or secondary) tumor resection on the rate of metastatic growth was ascertained for a wide variety of cancers and secondary sites:

- removal of a colorectal tumor accelerated growth of liver metastases [50];
- transurethral resection of prostate adenocarcinoma was found to promote metastasis [51];
- excision of melanomas precipitated very fast metastatic dissemination of the disease in three patients [52,53];
- resection of large metastases in eight patients with a particular kind of testicular cancer triggered a “sudden and dramatic exacerbation of the disease” [54].

Determination of the types of cancer and categories of cancer patients that fall within the scope of this striking phenomenon is clearly a *sine qua non* for understanding cancer and its treatment.

## 5. The Role of Mathematical Modeling

The above new paradigm of cancer can be formulated as the following hypotheses regarding the natural history of the disease and the effects of treatment:

- Metastatic dissemination may occur at early subclinical stages of cancer.
- Before the start of irreversible proliferation in a secondary site, metastasis may go through a protracted period of latency that includes free circulation, dormancy in the secondary site, slow avascular growth, and angiogenesis induction.
- Extirpation of the primary tumor promotes metastatic progression through interruption of metastasis dormancy, triggering vascularization of micrometastases, and acceleration of the growth of both avascular and vascular secondary tumors.

The above hypotheses are formulated in terms of events that are typically *unobservable* in an individual patient. This complicates their immediate application in cancer treatment. Another difficulty is that translating the novel cancer paradigm into an actual treatment plan requires quantification of the individual natural history of cancer. This amounts to determining, or estimating, the following quantities or parameters: (1) the age of onset of the primary cancer, that is, the time of the emergence of the first malignant clonogenic cell; (2) the time course of primary tumor growth; (3) the intensity of metastasis formation; (4) parameters characterizing the duration of metastasis latency (that is, the time between separation of a metastasis from the primary tumor and the start of its active irreversible proliferation in a host site including the times of free circulation, single cell dormancy, avascular growth, and angiogenesis induction); and finally, (5) the time course of metastasis growth at various secondary sites including the effects of treatment for the primary disease and metastases, unrelated medical interventions, and other relevant adversities.

How can one estimate these unknown and unobservable quantities? The key is to bridge them to *observable* clinical outcomes (such as cure, local tumor recurrence, surfacing of distant metastases or cancer-specific death). The difficulty here is that they are observed months, years and even decades after the occurrence of critical microevents that trigger natural or treatment-related processes that bring about these outcomes. The only feasible approach to building such a bridge is *comprehensive mathematical models* of the disease progression and treatment.

To estimate the multitude of unknown parameters related to the timing and rate characteristics of the individual natural history of cancer or effects of treatment, a mathematical model should be fit to the data available for the patient in question within a given clinical setting. Such observations typically fall into two categories: (1) clinical characteristics of the disease at presentation or primary diagnosis, and (2) observations that result from follow-up studies aimed at detecting primary tumor recurrence and/or metastatic spread of the disease. Observations in the first group usually include age at primary diagnosis; stage of the disease; localization and volume of the primary; its various histological, biochemical, and genomic characteristics; information on the familial history of the disease; and possibly the number, volumes, and sites of detectable metastases at presentation. Observations in the second group that are relevant to metastatic progression typically consist of information on the course of treatment for the primary tumor and metastases as well as the number, volumes, and sites of detectable metastases at certain time(s) post-treatment. Obtaining information on the number and volumes of detectable metastases with any reasonable accuracy is a daunting task, given various uncertainties associated with the reading of medical images, detection of metastases, and determination of their volumes. Some methodological approaches to estimation of the natural history of cancer are discussed in [55].

One of the greatest unknowns facing an oncologist who develops a treatment plan for a cancer patient is the possibility of the presence of *occult* metastases at presentation. In fact, the quality of treatment plan can be only as good as the information on their number and volumes. Once parameters of the comprehensive model of cancer natural history are estimated, the model can be utilized to assess the distributional characteristics of the number and volumes of occult metastases at any time point of interest. With this information at hand, one can design a plan of therapeutic intervention (including modes, dosage, and time courses of various treatments) that fits the patient best and maximizes the probability of cure or the residual lifetime.

## 6. Mathematical Model Selection

A large armamentarium of mathematical models applicable to cancer progression and treatment has been developed over the last 120 years. Which of them are suitable for validation of the novel paradigm of cancer, furthering our understanding of cancer dynamics, and designing better treatments? A few relevant criteria for model selection are discussed below.

### 6.1. Individual vs. Population Models

Many centuries of clinical practice have taught us that no two cancer patients are alike. While with some patients cancer can take an aggressive course, in other patients the same cancer may develop slowly and not be life threatening. Likewise, the same treatment may elicit immediate and long lasting positive response in one patient, have no effect on another, and initially show an excellent response only to be followed by aggressive relapse in yet another patient.

An immediate consequence of this individual manner of cancer progression and response to treatment is that preference should be given to *individual* models. The price one has to pay for such a selection is availability of only small set of values of clinical variables observed or measured for the patient in question that can be utilized for estimation of model parameters.

An alternative to individual cancer models is *population* models with some “averaged” values of model parameters assumed for the whole population. Such models can be fit to much larger data sets and hence have a greater statistical power for parameter estimation. However, such power may be undercut by heterogeneity of the population of patients to which the model is applied. The more homogeneous the population the more reliable and meaningful is the inference on model parameters.

One of the ways to mitigate this shortcoming of population models is to assume that model parameters are *random* and that their variation within the population is governed by a probability distribution from a certain parametric family. Naturally, this assumption expands the set of model parameters to be estimated from the data. Finally, feasibility of such an approach is typically sensitive to misspecification of the family of distributions employed to describe population heterogeneity with respect to parameters in question.

### 6.2. Mechanistic vs. Phenomenological Models

By the very nature of the new paradigm of cancer, any model employed to falsify its hypotheses (and generate new ones) should be formulated in terms of times of critical microevents (such as disease onset, shedding of metastases, and their inception at different secondary sites), durations of various processes (including primary tumor latency, free circulation of metastases, their avascular growth, and angiogenesis induction), rates of growth of primary and secondary tumors, concentrations of growth and angiogenesis factors and the corresponding inhibitors, *etc*. Therefore, these models are necessarily *mechanistic*. By contrast, *phenomenological* models establish quantitative relationships between observable explanatory variables (individual or population clinical covariates, treatment regimens and doses, and so on) and observable response variables (e.g. ages at clinical events, clinical variables, and treatment outcomes) based on correlations, likelihoods, regressions, and other statistical tools. Although such models may be useful for assessment of the effects of certain covariates, comparison of treatment strategies and the like, they of necessity play only an auxiliary role in ascertaining the validity of the new paradigm of cancer and establishing its limits. Another feature of phenomenological models that restricts their utility for understanding natural processes is that parameters of such models (e.g. regression coefficients) cannot be readily related to, or interpreted in terms of, biologically meaningful parameters. Finally, phenomenological models essentially establish correlations between input and output variables but cannot uncover cause-and-effect relationships.

### 6.3. Stochastic vs. Deterministic Models

Mathematical biologists have developed a plethora of deterministic and stochastic models for describing various processes associated with carcinogenesis, cancer progression, and treatment. For a fairly extensive overview of various deterministic models the reader is referred to [56,57]. Such models are based on ordinary or partial differential, integral or difference equations and can describe in great detail temporal and spatial dynamics of various biological processes implicated in cancer development and treatment, *inter alia* biochemical kinetics, transport processes, chemotaxis, cell motility, and angiogenesis. However, *comprehensive* models of carcinogenesis, cancer progression, and treatment are of necessity stochastic, for the following reasons:

- Accumulation of mutations in certain genes plays an important role in carcinogenesis. Such mutations are randomly occurring discrete events which cannot be modeled deterministically.
- Natural history of cancer is impelled through a number of sporadically occurring critical microevents [58] (emergence of the first clonogenic cancer cell, shedding of metastases and their inception in secondary sites, interruption of their dormancy, induction of angiogenesis, switching from avascular to vascular growth, *etc.*) and thereby is a stochastic process.
- Natural histories of cancer and responses to treatment involve substantial inter-subject and intra-subject variability in the genetic, physiological, and biochemical characteristics of primary and secondary tumors and their microenvironments, sensitivity to treatment, and other clinical variables. Naturally, stochastic models can account for a larger part of the observed variation of model outputs than deterministic models with the same outputs and of comparable complexity. Therefore, stochastic models are typically more *parsimonious* than their deterministic counterparts.
- The population of clonogenic cells in a naturally evolving, and especially treated, tumor can be quite small. Moreover, the outcome of treatment may be determined by survival of even a *single* clonogenic cell. Dynamics of such small populations can be described only probabilistically.
- Cell response to radiation involves quantum effects and various “hit-miss” contingencies that are inherently stochastic.
- Useful deterministic models have the following uniqueness property: the state of the process described by such a model at any moment of time is completely determined by its state at the initial moment of time. Complex biological systems and processes including cancer do not obey this law. Therefore, deterministic models are too rigid to account for the full extent of variability and uncertainty associated with cancer dynamics.

The dichotomy between deterministic and stochastic modeling is not absolute. Deterministic models of particular biological processes can be utilized as submodels within a “master” stochastic model. Also, differential equations can provide a mathematically tractable and computationally feasible description of the dynamics of the mean and variance of a random quantity, whose full stochastic dynamics is too complex to handle. Finally, solutions of differential equations may display chaotic behavior that captures certain important aspects of random processes.

### 6.4. Mathematical Models in Biomedical Sciences: Flexibility and Identifiability

Unlike universal laws in physics, regularities and patterns in biology are more flexible, subject to variation, and noisy. That is why comprehensive models of cancer natural history have to be based on a small number of fairly general assumptions and be minimally restrictive with regard to functional or distributional forms for the rates and durations of various biological processes. One of such models is described in detail in Section 7.

Another fundamental problem associated with modeling biomedical processes is specification of model parameters. While in physics and chemistry these parameters are often “universal” and can be estimated or measured once and for all, in biology and medicine they are widely varying between species, tissues, cell types, experimental conditions, and even individuals; also, they may change in time. Furthermore, parameters measured *in vitro* typically bare little relevance to those *in vivo*. Thus, utility of mathematical models of biomedical processes is inseparable from our ability to reliably estimate model parameters. As a result, every model should be tailored to the size, structure, and quality of a data set intended for parameter estimation purposes. An exception is (fairly rare) general models whose outputs have universal qualitative features (such as monotonicity, uni- or bimodality, *etc.*) regardless of particular values of model parameters, which would warrant using such models for testing biomedical hypotheses.

The link between models and samples of their observed outputs employed for parameter estimation imposes limits on model complexity. Although increasing the number of model parameters will improve the fit to the data, at some point it will render the model *non-identifiable* [59]. Such is a model that, by definition, produces identical outputs for distinct sets of model parameters. Model identifiability, then, is an intrinsic structural property of the model. Although this property can at times be established analytically [59], it invariably manifests itself when parameters of the model are being estimated. Theoretically, parameters of a non-identifiable model are inestimable from a sample of observations of the model output. In practice, an attempt to statistically estimate model parameters without regard for non-identifiability would lead to instability of the estimates. Therefore, non- identifiable models have limited utility for furthering and quantifying our knowledge about various biomedical processes, including cancer progression and treatment.

It can be shown that model non-identifiability can be lifted through reparameterization [59]. However, new identifiable parameters represent only combinations of the original biological parameters from which the latter cannot be recovered uniquely. Another means of making a model identifiable is to expand the set of model outputs and, accordingly, observable variables used for parameter estimation. In summary, preference should be given to identifiable models that provide a reasonably good fit to data, thus striking a delicate balance between biomedical adequacy, mathematical tractability, and statistical feasibility.

## 7. A Model of Individual Natural History of Metastatic Cancer Accounting for the Effects of Surgery

In this section, we describe a comprehensive, entirely mechanistic, stochastic, parsimonious model of individual natural history of disseminated breast (or any other solid) cancer developed in [60,61]. The output of the model is the distribution of the volumes of metastases in a given secondary site. The model was applied in [62] to the observed volumes of bone metastases in a particular breast cancer patient, which enabled estimation of the most important parameters descriptive of the patient's natural history of breast cancer and the effect of surgery on the rate of growth of metastases. The results of the analysis, reviewed in what follows, provide a definitive corroboration, for the patient at hand, of all the hypotheses of the novel paradigm of cancer outlined in Section 5.

### 7.1. Model Description

The temporal natural history of invasive cancer consists of three components: primary tumor latency, primary tumor growth, and metastatic progression. The latency period starts with the birth of an individual (or start of exposure to a carcinogen in case of induced carcinogenesis) and ends with the appearance of the first malignant clonogenic cell, termed the *onset of the disease*. These components and relevant model assumptions are described below.

1. *Primary tumor growth.* It is assumed that primary tumor originates from a single clonogenic cell and that the tumor size (that is, the number of cells comprising the tumor) at time t counted from the onset of the disease, Φ(t), is an *arbitrary* increasing continuous function that satisfies the condition Φ(0) = 1 and may depend on one or several parameters.
2. *Metastasis formation.* The main driving force behind the model is the assumption (introduced in [60], see also [63]) that the process of metastasis shedding by the primary tumor is governed by a Poisson process whose rate μ is proportional to some power of the current size of the primary tumor:
  (1) $$\mu \left(\text{t}\right){=\alpha \Phi }^{\theta }\left(\text{t}\right),$$
  where α > 0 and θ ≥ 0 are constants. For θ = 0 the Poisson process is homogeneous, in which case the rate of metastasis shedding is independent of the primary tumor size. Parameter θ is descriptive of the fraction (and, indirectly, spatial distribution) of primary tumor cells with metastatic potential. The value θ = 1 suggests that all cells in the primary are potentially metastatic. If cells with metastatic potential cluster close to the tumor surface, one would have θ = 2/3. Incidentally, the estimate of the fractal dimension of blood vessels that serve as a conduit for bloodborn metastases is also close to 2/3 [64]. Finally, small θ would indicate the presence of a small reserve of highly metastatic tumor cells.
  It is further assumed that metastases shed by the primary tumor give rise to clinically detectable secondary tumors in a given site independently of each other with the same probability p. According to one of the basic properties of Poisson processes, this implies that inception of metastases, which will eventually become detectable secondary tumors in the site in question, is also governed by a Poisson process with the rate pμ(t). Each such viable metastasis is assumed to spend some random time between detachment from the primary tumor and the start of its irreversible vascular growth in a secondary site, termed *metastasis latency time*. We assume that latency times for different metastases are independent and identically distributed random variables with absolutely continuous distribution. Then, as shown in [65], emergence of viable metastases in the secondary site again follows a Poisson process.
3. *Metastasis growth.* Our main premise is that prior to primary tumor resection the growth of any viable metastasis in a given site is governed by a function ψ_0_(t), where time t is counted from the end of metastasis latency period, while after the resection, the size of metastases is growing according to another function ψ_1_(u), where u is time since surgery, such that ψ_1_(0) =1. Because the size attainable by an avascular secondary tumor is small [66] and its growth is slow, we assume for simplicity that ψ_0_(0) = 1. Functions ψ_0_ and ψ_1_ are increasing and differentiable but otherwise *arbitrary*. Additionally, they may depend on one or several parameters. A simpler setting where ψ_1_ = ψ_0_, *i.e.*, no effect of surgery on metastatic growth rate, was explored in [60].
4. *Secondary metastasis.* We suppose that secondary metastasizing (that is, formation of “metastasis of metastasis”) to a given site, both from other sites and from within, is negligible.
5. *Metastasis detection.* A metastasis becomes detectable when its volume reaches some threshold value m determined by the sensitivity of imaging technology. In the case of PET/CT imaging involved in the study [62], m = 0.5 cm^3^, and the accuracy of volume determination is one voxel, *i.e*., approximately 0.065 cm^3^.
6. *Effects of treatment*. Because the rate of secondary metastasizing is assumed negligible, the process of metastasis formation is stopped at the time of extirpation of the primary tumor. Adjuvant chemotherapeutic, hormonal or other treatment is assumed to affect metastases only through the rate of their growth (and not through prolongation of their latency times).
7. *Timeline and observables*. The age at primary diagnosis is denoted by U and the primary tumor volume at diagnosis by S. It is supposed also that at age V, V ≥ U, the primary tumor was resected, and that at age W, W ≥ V, the patient developed n detectable metastases in the same secondary site with the observed volumes X_1_, X_2_,…, X_n_, where X_1_ < X_2_ < … < X_n_.

### 7.2. Distribution of the Volumes of Detectable Metastases

Due to the non-stationarity of the process of metastasis shedding by the primary tumor, the volumes of metastases detectable in a given secondary site at a given time post-diagnosis do not form a random sample from a probability distribution. However, it was shown in [60,61] that the volumes of metastases are *equidistributed*, conditional on their number n, with a random sample of size n from a certain distribution, and its probability density function (pdf) f was computed explicitly in [61,62]. This function depends only on the law of primary tumor growth, Φ; the pre-surgery and post-surgery laws of growth of metastases, Ψ_0_ and Ψ_1_; parameter θ involved in formula (1); the distribution of metastasis latency time; and the observables. Setting the detection threshold m equal to the volume of one cell produces the distribution of the site-specific volumes of *all* (both occult and detectable) metastases.

The above equidistribution property, that essentially depends on the assumption that metastasis shedding is a Poisson process described by formula (1), opens up a possibility of estimating the natural history of cancer and the effect of surgery on the rate of metastasis growth. In fact, the distribution of any rearrangement-invariant statistic based on the observations X_1_, X_2_,…, X_n_ (such as sample mean or variance) would be identical to the distribution of the same statistic based on a random sample of size n drawn from the pdf f. In particular, the joint likelihood of the observations X_1_, X_2_,…, X_n_,

$$\text{L}({\text{X}}_{1},{\text{X}}_{2},\ldots ,{\text{X}}_{\text{n}})=\text{n}!\prod_{i=1}^{n}f(X_{i}),$$

has the same form (apart from the factor n!) it would take should the observations X_1_, X_2_, …, X_n_ form a random sample from the distribution with pdf f. Therefore, identifiable parameters of the suitably parameterized model can be estimated using the method of maximum likelihood.

### 7.3. Model Parameterization

To enable quantitative inference on the natural history of cancer and the effects of surgery, the above general model of cancer progression was parameterized. Specifically, it was assumed that the primary tumor grows exponentially with rate β: Φ(t) = e^βt^. Similarly, metastases were assumed to grow exponentially with a pre-surgery rate γ_0_ and post-surgery rate γ_1_. Finally, metastasis latency times were supposed exponentially distributed with the expected value ρ, so that their pdf has the form ρ^−1^e^−t/ρ^.

The resulting parametric model depends on the following seven parameters: β (the rate of growth of the primary tumor); α and θ (two parameters involved in the expression (1) for the Poisson rate of metastasis shedding); p (the probability that a cancer cell shed by the primary tumor will give rise to a viable metastasis in a given secondary site); γ_0_ (the rate of growth of metastases in the presence of the primary tumor); γ_1_ (the rate of growth of metastases after its excision); and ρ (the mean metastasis latency time). However, pdf f of the distribution underlying the site-specific volumes of metastases, see Section 7.2, depends only on five parameters β, θ, γ_0_, γ_1_, ρ and does not involve parameters α and p, see [61,62] for an explicit expression. These five parameters are identifiable from the observed site-specific volumes of metastases [60,61], provided that the volume of the primary and metastases are surveyed at distinct times. To estimate the values of model parameters, we fit the model to a particular data set.

### 7.4. Data Analysis

We used a data base of several hundred breast cancer patients who were diagnosed and treated at the Memorial Sloan-Kettering Cancer Center (MSKCC). The treatments consisted of various combinations and time courses of surgery and adjuvant radiation-, chemo-, and hormonal therapies. To enable application of the above model, a patient had to meet several requirements: (1) treatment includes excision of the primary tumor (mastectomy or lumpectomy); (2) the whole body PET/CT scans are available; (3) the number of metastases in a single secondary site is large enough; (4) primary tumor volume at presentation is available; and (5) no change of treatment occurs between primary diagnosis and metastasis surveying, which would allow us to apply the above parametric model with a single set of kinetic parameters β, θ, γ_0_, γ_1_, ρ. Additionally, to ensure identifiability of the entire set of model parameters, the volumes of the primary tumor and metastases have to be measured at different times.

Among all the patients in the data base, only one patient satisfied all the above conditions. At age U = 74 years she was diagnosed with stage III estrogen receptor positive primary breast cancer, and the volume of the primary tumor at presentation was S = 10.3 cm^3^. Shortly after the diagnosis, the patient underwent radical surgery (U = V = 74 years) and was put on adjuvant hormonal therapy with tamoxifen. At age W = 82 years (more exactly, eight years and five days after the primary diagnosis), 37 bone, lung, lymph node, and soft tissue metastases were discovered. Volumes of all the metastases were measured through laborious reading of the whole body PET/CT scans. In particular, the volumes of n = 31 bone metastases were:

1.69, 1.98, 2.01, 2.04, 2.14, 2.20, 2.46, 3.05, 3.18, 3.31, 3.37, 3.48, 3.52, 3.57, 4.22, 4.34, 4.73, 5.04, 5.08, 5.25, 5.45, 5.64, 6.36, 6.55, 7.39, 9.01, 9.21, 11.15, 12.71, 13.81, 22.96 cm^3^.

Additionally, the patient developed three lung metastases with the volumes 1.30, 2.01 and 7.26 cm^3^, two lymph node metastases with the volumes 2.85 and 9.66 cm^3^, and one soft tissue metastasis with the volume 11.41 cm^3^.

Model parameters were estimated from the volumes of bone metastases by the method of maximum likelihood. For conversion of tumor volume into tumor size, we assumed that 1 cm^3^ of primary or secondary tumor contains 10^9^ cells, a standard assumption for most solid tumors. The resulting parameter estimates are:

$$\beta =0\text{.717}{\;\text{years}}^{-1},\theta =0.025,\gamma_{0}=0.083{\;\text{years}}^{-1},\gamma_{1}=2.676^{-1},\rho =79.52\;\text{years}\text{.}$$

The model-based cumulative distribution function (cdf) corresponding to pdf f, that determines the distribution of the volumes of bone metastases given their number n = 31, with optimal parameters provided an almost perfect fit to the empirical cdf [62]. Finally, estimates of model parameters were found to be fairly insensitive to small errors (up to one voxel) in determination of the volumes of metastases.

### 7.5. Model Validation

It could be argued that establishing data fitting power of the model for a single patient is insufficient to claim model adequacy in general. Also, one could attribute the observed excellent fit to the data to the effects of overparameterization, *i.e.*, the ability of our 5-parametric model to fit any regular enough data set. To address these two concerns, we analyzed data sets for two additional breast cancer patients from the MSKCC data base. One of these data sets satisfied requirements 1–5 formulated in Section 7.4. However, in both cases the model experienced a considerable degeneration (into three- and two-parametric models), which prevented us from estimating all relevant model parameters. Note that even in these cases the model remains fully specified by the reduced set of identifiable parameters making it possible to optimize them in the maximum likelihood setting and assess how well the degenerate models fit the observed volumes of bone metastases. We found that, in spite of the degeneration, the model still fits the data very well and, furthermore, the values of estimable model parameters for these two additional patients are in good agreement with those found for the main patient [62].

### 7.6. Model-based Reconstruction of the Individual Natural History of Cancer

Knowledge of the estimates of all identifiable model parameters allowed us to describe, for the main breast cancer patient, the principal timing and rate characteristics of the natural history of the disease and the effects of surgery on the growth of bone metastases.

The onset of the disease occurred at age 42, about 32 years prior to the detection of the primary tumor. The progression of the primary breast cancer was quite slow with the tumor doubling time of about 11.5 months. The inception of the first bone metastasis (which, according to the model, is the one with the largest volume at detection) occurred at the age of 44.5, about 29.5 years prior to the primary diagnosis, at which time the primary tumor consisted of just a handful of cells. The growth of bone metastases prior to extirpation of the primary tumor was extremely slow with the doubling time of about 8.3 years. Resection of the primary tumor was followed by a 32-fold increase in the rate of growth of metastases with an equivalent drop in the doubling time to 94.5 days. The mean metastasis latency time was very long, about 79.5 years.

### 7.7. Results of Mathematical Modeling and the New Paradigm of Cancer

The reconstructed natural history of cancer for our main breast cancer patient provides a definitive confirmation of all the postulates of the new paradigm of cancer. It also agrees with other well-known findings regarding the natural history of breast cancer, and may help generate new hypotheses and directions of scientific inquiry. We summarize our findings:

1. The onset of breast cancer was a very early event in the natural history of the disease: it occurred at age 42, about 32 years prior to the primary diagnosis.
2. The model produced a very small value of parameter θ in all three patients analyzed. This means that, first, the process of metastasis shedding by the primary tumor is essentially homogeneous in time; and, second, that only a small fraction of cells in the primary has high metastatic potential.
3. Dissemination of metastases was an extremely early event in the natural history of the disease. In fact, according to the model, inception of the first bone metastasis occurred 2.5 years after disease onset and about 29.5 years before primary tumor detection (shedding of this metastasis by the primary tumor occurred even earlier). At that time, the primary tumor was so small that extrapolation of any deterministic law of tumor growth for estimation of its size would be misleading.
4. At the time of primary tumor resection, all metastases that have reached their target secondary site (the skeletal system) were extremely small and growing very slowly to the extent they could be called dormant for all practical purposes. Therefore, the model decidedly supports the notion of metastasis dormancy.
5. The very large value of the mean latency time for bone metastases (ρ = 79.5 years) suggests that, at the time of metastasis surveying (eight years after primary diagnosis), most bone-bound metastases either circulated in blood or lymph vessels or remained dormant in the bones. However, pooling together the volumes of all 37 metastases regardless of their sites led to a more than 15-fold decrease in the mean metastasis latency time (ρ = 5.2 years). This suggests that (a) metastasis latency time is remarkably site-specific; and (b) dormancy of metastases in their target sites is prevalent over free circulation.
6. The most striking finding of our analysis is the 32-fold increase in the rate of post-surgery metastasis growth, as compared to the pre-surgery rate. Given that after surgery the patient was put on adjuvant therapy with tamoxifen, which suppresses growth of estrogen-dependent tumors and also has anti-angiogenic activity, this effect is likely to be even more dramatic, were the patient not treated with tamoxifen. It is important to emphasize that surgery-driven acceleration of the growth of metastases was not assumed by the model but rather inferred from the data through estimation of model parameters.
7. Metastatic potential of the primary tumor depends largely on parameters αp that represent the rates of formation of viable metastases at various secondary sites per one clonogenic cell of the primary tumor, see Section 7.1. Although the quantity αp does not enter the distribution of the volumes of metastases in a given site, it determines the distribution of their *number*. Estimation of parameter αp for the secondary site of interest requires availability of longitudinal data on the number of detectable metastases in this site surveyed at several time points.

### 7.8. Model Refinement

It could be argued that the model of cancer natural history employed in our analysis is not detailed enough to capture the true complexity of biomedical processes it seeks to describe. To a large extent, the complexity of the model is dictated by the extent of the set of observables available for an individual patient. Within these constraints, the model not only provided an excellent fit to the data but also proved identifiable, which allowed us to estimate the most important parameters descriptive of the natural history of breast cancer and the effect of surgery on the rate of growth of bone metastases. As more clinical data become available (such as larger numbers of metastases at various secondary sites or individual longitudinal data on metastasis volumes surveyed at several time points), the model can be refined to accommodate them and provide a more detailed picture of cancer natural history, at the expense of expanding the set of model parameters. A number of such refinements are readily envisioned:

- The exponential laws of growth of the primary tumor and metastases can be replaced with more realistic Gompertz, or other, laws.
- The duration of primary tumor growth can be broken into the periods of slow avascular growth, angiogenesis induction, and growth of the vascularized tumor, each with its own rate parameters. Similarly, the metastasis latency period can be partitioned into sub-periods of free circulation, single cell dormancy, avascular growth, and angiogenesis induction, with their own kinetic characteristics.
- The model may postulate two distinct metastasis latency times—one before and one after the removal of the primary tumor—and thus incorporate the effects of surgery on the duration of metastasis latency.
- The model can be expanded to encompass the effects of radiation and chemotherapy on the dynamics of metastasis latency and growth.

## 8. Some Methodological Pitfalls

It became customary to lard biomedical literature with all kinds of statistical indicators (p-values, correlations, confidence intervals, *etc*.) without paying any attention to the assumptions on relevant variables that a particular indicator entails. Such assumptions typically include, but are not limited to, independence and identical distribution of the variables involved, normality, and large sample size. In many cases such assumptions are not met even by very liberal standards. As a result, conclusions based on such an abuse of statistics run a risk of producing false knowledge.

Quite often, arbitrary sets of observations or measurements are viewed as “random samples” and treated with an assortment of statistical methods. The model discussed in Section 7 presents lucidly a pitfall of such an indiscriminant approach. As discussed in Section 7.2, the observed volumes of metastases in a particular site do not form a random sample from any probability distribution because these volumes cannot be viewed as resulting from measurements that are repeated independently under identical conditions. In fact, the volumes of detectable metastases are dependent random variables and have different distributions. A concomitant problem is the lack of a “natural” order in listing these volumes; when listed in an increasing (or decreasing) order, they definitely become dependent and forced to have distinct distributions. Fortunately, this was mitigated by the fact that the volumes of metastases are *equidistributed* with a random sample from a computable distribution, which enabled utilization of rearrangement-invariant statistics for parameter estimation purposes. However, this possibility is only due to the fact that the times of occurrence of the events of interest (*i.e.*, shedding of metastases by the primary tumor) are assumed to follow a Poisson process.

As biomedical sciences become increasingly quantitative, their emerging hypotheses tend to be tested more often on the basis of mathematical models. The data required for estimation of model parameters are generated through biological experiments and clinical observations, which are at times sophisticated, time consuming, and costly. To be useful, they should produce data whose structure and quantity satisfy model assumptions, be commensurate with model complexity, and render model parameters identifiable. That is why biomedical scientists should consult mathematicians and statisticians at the *planning stage* of their data collection effort, rather than after the data have been collected.

## 9. Conclusions

Profuse evidence accumulated over several recent decades points to the fact that the traditional paradigm of cancer based on genetically driven, linear progression of cancer is inconsistent with the totality of clinical experience, epidemiological data, results of the experiments on animals, and conclusions drawn from mathematical models. The emerging new paradigm of cancer proposed in [12,20] and explicated in the present work represents a working hypothesis that is not only compatible with such evidence but also provides compelling grounds for numerous biomedical observations that have evaded alternative explanation. The core of the new paradigm of cancer is four-fold: (1) early systemic spread of cancer; (2) prevalence of cancer dormancy; (3) importance of angiogenesis as a critical step in cancer progression; and (4) surgery-induced acceleration of the growth of metastases and their vascularization. This suggests radically new ways of cancer control and treatment, including maintaining the state of cancer dormancy. A critical impediment to validation of the novel theory of cancer is that it deals with the events that are typically unobservable in an individual patient. Such validation can be effectively done through comprehensive mathematical models of cancer natural history and the effects of cancer treatment. Additionally, such models can facilitate comparison *in silico* of various treatment scenarios. The biomedical community has to wake up to the uncovered reality of cancer. Should it continue along the beaten track, the victory in the war on cancer will remain elusive.
